# Flexural Strengthening of RC Slabs with Lap-Spliced Carbon Textile Grids and Cementitious Grout

**DOI:** 10.3390/ma15082849

**Published:** 2022-04-13

**Authors:** Hyeong-Yeol Kim, Young-Jun You, Gum-Sung Ryu

**Affiliations:** Structural Engineering Department, Korea Institute of Civil Engineering and Building Technology (KICT), Goyang-Si 10223, Korea; hykim1@kict.re.kr (H.-Y.K.); ryu0505@kict.re.kr (G.-S.R.)

**Keywords:** carbon textile, cementitious grout, concrete structure, flexural strengthening, textile-reinforced concrete (TRC), fabric-reinforced cementitious matrix (FRCM), structural testing

## Abstract

This paper presents a new textile-reinforced concrete (TRC) installation method for strengthening structurally deficient or damaged reinforced concrete (RC) structures with grouting. In this study, cementitious grout was used as a matrix for the TRC system. TRC coupon specimens with different lap-splice lengths were tested under tension to determine the minimum textile lap-splice length. The minimum lap-splice length of the sand-coated textile was evaluated as 150 mm. The performance of the TRC-strengthened RC slabs with the proposed installation method. The lap-spliced textile was experimentally validated by a flexural failure test. Five RC slabs were strengthened by one ply of sand-coated carbon textile grid with and without the lap-splicing and 20 mm-thick cementitious grout and were tested in flexure. Among the TRC-strengthened RC slab specimens, two specimens were re-strengthened RC slabs with the TRC system. The TRC strengthened slab, for which the lap-splice length of the textile was 50% smaller than the minimum lap-splice length, failed at the load level of steel yield. On the other hand, the ultimate load-carrying capacity of the RC slabs strengthened by the TRC system with textile lap-splicing decreased by at least 6% relative to that without textile lap-splicing. Furthermore, the results of a flexural test for the TRC re-strengthened slabs indicate that the ultimate load-carrying capacity of the TRC re-strengthened slabs is almost the same as that of an undamaged slab strengthened with the TRC system.

## 1. Introduction

Structurally deficient reinforced concrete (RC) structures should be adequately strengthened with efficient as well as effective materials. Among the existing materials for the strengthening of RC elements, textile-reinforced concrete (TRC) has widely been recognized as an ideal material due to its light weight, high load-bearing capacity, high durability, and ease of installation.

Textile reinforcements can be fabricated in two- or three-dimensional shapes, a two-dimensional grid type of textile has been commonly used for the strengthening of RC structures [1,2,3]. In general, a textile grid is integrated with concrete or mortar to form a TRC system. If mortar is used, we refer to it as a textile-reinforced mortar (TRM) system [4].

In the previous experimental studies conducted on the strengthening of RC structures with a TRC system, structurally deficient RC beams were strengthened by a TRC system in flexure [5,6,7,8,9] and in shear [10]. Textile coating [5], textile anchors [6,10], and textile pre-tensioning [7] were generally considered as design variables in earlier experimental studies. Other design variables that have been considered are the matrix composition [8] and the number of textile plies [9,10]. The findings of these studies verify that the strength of the matrix has an insignificant influence on the effect of strengthening.

Numerous research groups conducted studies into the strengthening of RC slab elements with TRC systems [11,12,13,14,15]. RC slabs were strengthened in flexure by a carbon textile [11] and a PBO textile [12]; their studies confirmed that the behavior of a TRC-strengthened RC slab could be estimated by analytical calculation. More recently, RC slab elements were strengthened with a carbon TRC system [13,14] and with a precast TRC panel [15]. The results of these studies demonstrate that, regardless of the installation method, a TRC system can effectively be used for the strengthening of deteriorated or structurally deficient RC elements.

**Figure 1:**
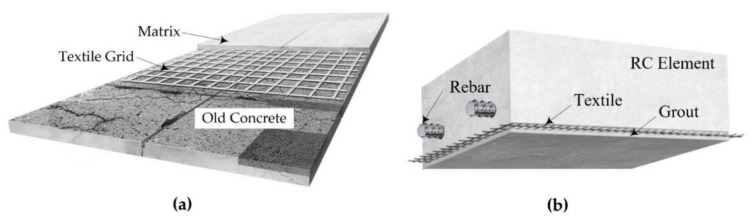
TRC-strengthening methods for concrete elements: (**a**) overlay method; (**b**) proposed method.

A direct application of the TRC system by hand lay-up or a spraying method over the surface of existing RC structures is a commonly utilized on-site TRC installation method (Figure 1a). An on-site grouting TRC installation method (Figure 1b) was proposed for RC structures with difficult accessibility or with a narrow working space [16]. In the proposed installation method, the textile grid is first fixed to the existing concrete structure to be strengthened, and then conventional formwork is assembled over the textile grid. Finally, TRC strengthening is completed by filling a cementitious grout into the space between the existing concrete and preassembled formwork.

One of the issues regarding the installation of a TRC system in practice is the lap-splicing of the textile grids. The textile grid generally requires on-site lap-splicing since the size of commercially available textile grids is generally limited. The lap-splice length of the textile grid must be longer than the minimum lap-splice length of the textile grid to safely transfer the tensile stress of the lap-spliced textile. Note that the minimum lap-splice length of the textile can be evaluated by a direct tension test such as that specified in [17]. 

Another issue dealing with the TRC strengthening of RC elements is the bond behavior between concrete and the TRC system. Recently, pull-off tests were directly conducted on the surface of the TRC system cast on an RC slab-type element to examine the bond behavior of the TRC system [18]. The test results of this study confirm that the predominant failure mode was a tensile failure of the concrete substrate rather than an interfacial failure. Furthermore, the influence of environmental conditions (humidity and temperature) on the bond behavior of PBO grid and cement-based mortar has been studied [19,20]. The results of these studies indicate that the TRC system is not sensitive to humidity and temperature.

This study deals with flexural tests of full-scale RC slabs strengthened with lap-spliced textile grids. The objectives of this work are to examine the tensile performance of a TRC system with lap-spliced textiles and to validate the structural performance of RC slabs strengthened with lap-spliced textiles. If the proposed method of TRC installation is successfully developed, the TRC strengthening of RC structures with difficult accessibility can be easily conducted. Furthermore, if the proposed method is developed, textile lap-splicing can be easily performed during the TRC installation.

TRC coupon specimens were fabricated with two different types of carbon textile grids (sand-coated and uncoated textiles) and a cementitious grout. Full length and two different lap-splice lengths of textile were considered for the fabrication of TRC specimens to examine the influence of textile lap-splice length on the tensile behavior of the TRC system. Eighteen coupon specimens, nine for each textile grid, were tested in tension. The tensile behavior of the lap-spliced specimens was compared with that of a specimen with full-length textile.

A total of five 2000.0 mm-long, 500.0 mm-wide, and 200.0 mm-thick full-scale RC slabs were strengthened in flexure with a carbon TRC system (20.0 mm-thick): one has a full-length textile with no lap splice; four have a lap-spliced textile at the middle of the slab. The textile grid used for the TRC strengthening is the grid that showed better tensile performance in the direct tensile test. Among the TRC-strengthened RC slab specimens, two of the specimens were prepared by re-strengthening damaged RC slabs that were failure tested in a previous study [16]; therefore, another objective of this study is to examine and validate the effectiveness of the TRC-strengthening method for damaged RC elements where steel reinforcement completely yielded.

The RC slabs were strengthened by an on-site TRC grouting installation method and were tested by a three-point bending test until failure to identify the influence of textile lap splice on the load-carrying capacity. The structural performance of the TRC-strengthened RC slabs was compared with the results of a previous study [16] and of an unstrengthened RC slab.

## 2. Tensile Behavior of TRC System with Lap Splice

### 2.1. Materials

In this study, the tensile behavior of a TRC system with lap-spliced textile was investigated by a direct tension test [17]. Two types of carbon grids were used as textiles (Table 1). Table 2 provides the mix composition of the cementitious non-shrink grout that was used as a binder for the TRC system. The design strength of the grout is 50 MPa.

### 2.2. Fabrication of Tensile Test Specimens

**Figure 2:**
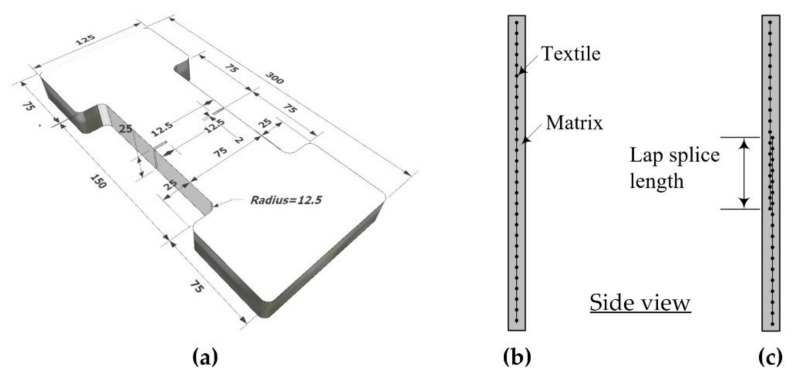
Coupon specimen for direct tension test: (**a**) dimensions (units, mm); (**b**) coupon without textile lap splicing; (**c**) coupon with lap splicing.

Figure 2a shows a dumbbell-type coupon specimen used in the direct tension test. Figure 2b,c, respectively, show a side view of the specimen with and without a textile lap splice. Note that the thickness of the tension test specimen is selected as 25 mm, which is similar to the thickness of the TRC system to be used in the strengthening work for RC slabs. 

Table 3 provides the characteristics of the two groups of specimens used in the test. Textile grid type and lap-splice length ($L_{LS}$) were considered as the design variables. In the test, the maximum lap-splice length was set to 150 mm. Note that the length of the load transition zone of the specimen is 150 mm (Figure 2a). The minimum lap-splice length of textile recommended in the design guideline [17] is 51 mm.

**Figure 3:**
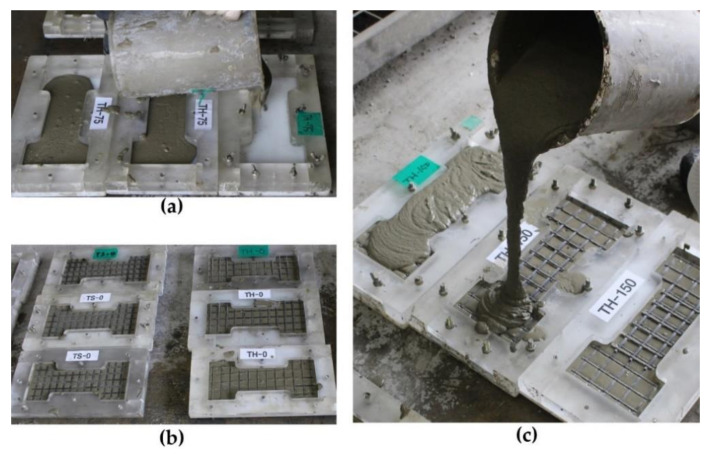
Fabrication process of tension test specimens: (**a**) placement of 1st layer of grout; (**b**) placement of textile grid; (**c**) placement of 2nd layer of grout.

Figure 3a,c shows the fabrication process of the dumbbell-type coupon specimen with a plastic mold. As illustrated in Figure 3b,c, the textile grids were placed at the mid-plane of the grout layer. The coupon specimens were cured with a plastic covering in a temperature-controlled room at about 20 °C for 28 days. 

### 2.3. Results of Tensile Tests

**Figure 4:**
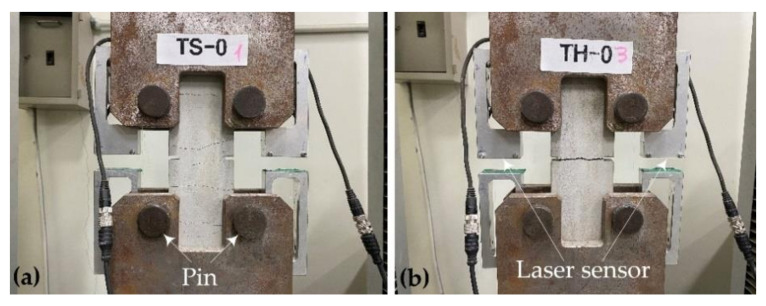
Test setup, instrumentation, and failure mode: (**a**) TS-0 specimen; (**b**) TH-0 specimen.

**Figure 5:**
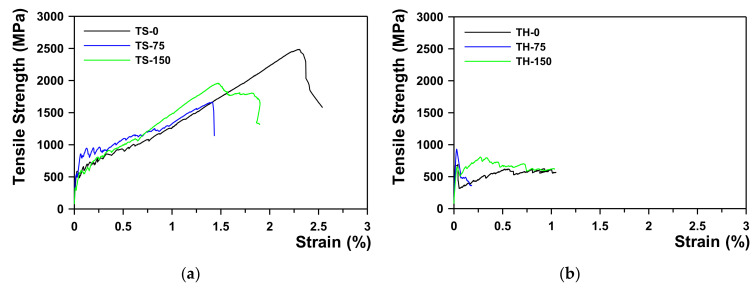
Axial stress–strain curve of tension test specimens: (**a**) TS series; (**b**) TH series.

Figure 4a,b shows the test setup and failure mode for the TS-0 and TH-0 series specimen, respectively. The results of the direct tension tests are summarized in Table 4. Figure 5a,b shows the axial stress–strain curve of the TS and TH series specimens, respectively.

The ultimate tensile strength ($f_{fu}$) of the specimens was greatly influenced by the type of textile and the lap-splice lengths. The tensile tests revealed that the direct tensile strength of the sand-coated textile without a lap splice (TS-0 specimen) was 2112.5 MPa and the ultimate tensile strain ($\varepsilon_{fu}$) was 1.912%. For $L_{LS}$ of 75 mm and 150 mm, the average value of $f_{fu}$ was 1689.3 MPa and 2169.3 MPa, respectively, and that of $\varepsilon_{fu}$ was 1.447% and 1.716%, respectively. Compared to the TS-0 specimen, $f_{fu}$ was about 80% when $L_{LS}\;$ was 75 mm, and 100% $f_{fu}$ was achieved when $L_{LS}$ was increased to 150 mm; therefore, the minimum $L_{LS}$ of TX-1 can be evaluated as 150 mm by this test. In addition, in the stress–strain behavior of textile, as the bonding strength with the matrix was enhanced due to sand-coating on the textile surface (TS series), strain-hardening occurred, where the stress increased along with increases in the strain after initial cracking, regardless of the lap-splice length. 

However, when the textile surface was uncoated (TH series) and not lap spliced, $f_{fu}$ was 923.5 MPa, and for $L_{LS}$ 75 mm and 150 mm, it was 961.3 MPa and 912.9 MPa, respectively, indicating that $L_{LS}$ did not significantly affect $f_{fu}$; therefore, the minimum $L_{LS}$ of TX-2 could not be correctly evaluated by this test. In addition, the stress–strain behavior of the TH series showed strain-softening due to a sudden stress drop after initial matrix cracking. This is presumably because the bonding strength between the textile and the matrix failed to resist after initial cracking, causing the slip. 

The first cracking strength ($f_{cr}$) of the TRC system was at most 4.1 MPa regardless of the specimen types. For the TS specimens, strain-hardening occurred as the stress was transferred after initial cracking at the center, resulting in several subsequent cracks over the coupon specimen (Figure 4a). The failure mode of TS specimens indicated that the induced tensile stress was well distributed along the coupon specimen with the sand-coated textile. As for the TH specimens, however, only a single crack occurred, and this is because the stress could not be transferred as the textile slipped due to reduced bonding strength between the textile and the matrix after initial cracking at the center (Figure 4b). 

## 3. Flexural Test for TRC-Strengthened RC Slabs

### 3.1. Fabrication of RC Slabs

**Figure 6:**
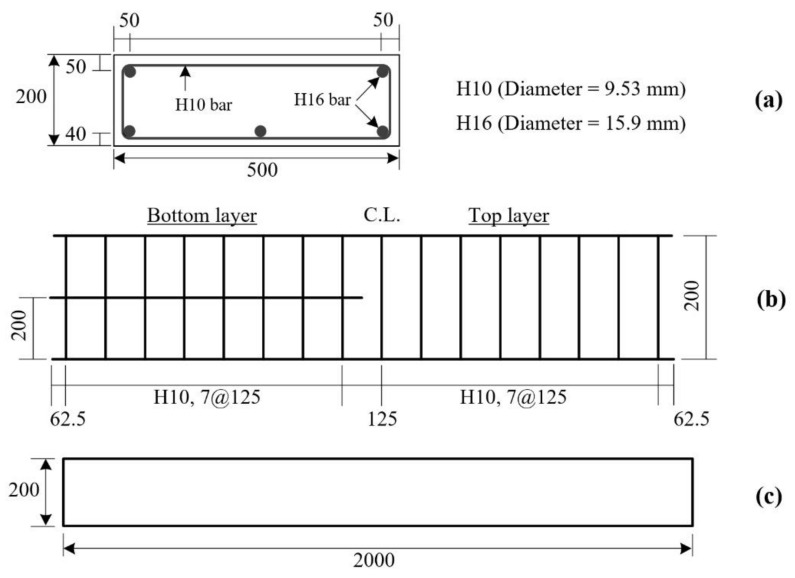
RC slab: (**a**) cross-sectional dimensions; (**b**) reinforcement details; (**c**) side view (unit: mm).

First, three 500.0 × 200.0 × 2000.0 mm^3^ (width × height × length) RC slabs were fabricated. Figure 6a,b, respectively, illustrate the dimensions and steel reinforcement arrangement of the RC slab. On the other hand, Figure 6c shows a side view of the RC slab. The mix composition of the concrete used is summarized in Table 5. The yield strength of the 16 mm bar and 10 mm bar was 451 MPa and 488 MPa, respectively. Note that, in this study, the RC slab is designed as an under-reinforced slab to maximize the TRC-strengthening effect. A practical approach and numerical procedure for estimating the deformability of RC slabs in buildings can be found in the literature [22,23].

### 3.2. TRC Strengthening of Slabs

**Figure 7:**
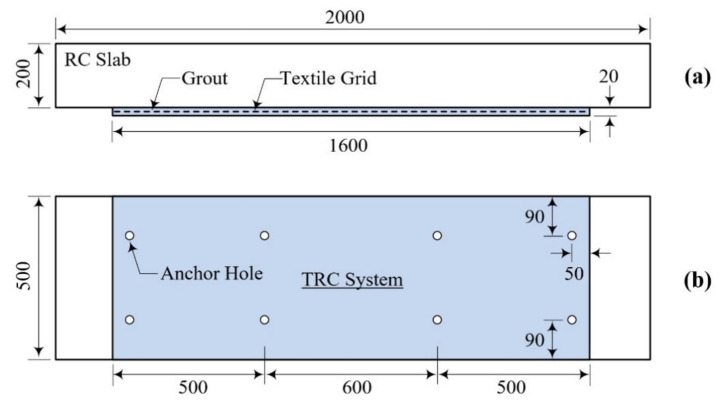
TRC-strengthening plan for RC slab: (**a**) side view; (**b**) bottom view (unit: mm).

**Figure 8:**
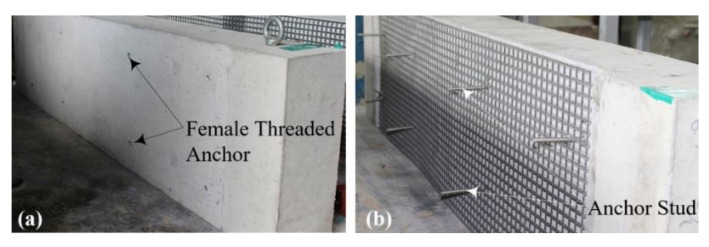
TRC-strengthening process for RC slab: (**a**) grinding and installation of the female-threaded anchor; (**b**) installation of textile grid and male-anchor studs.

Figure 7a,b illustrates TRC-strengthening plans for the RC slabs. As shown in Figure 8a, the region underneath the RC slab was directly strengthened by the TRC system. All slab specimens were strengthened with a single ply of the sand-coated textile grid (Tx-1). The Tx-1 grid was selected for the TRC strengthening because it showed better tensile performance than Tx-2 in the direct tensile test. The binder used for the TRC system was a cementitious non-shrink grout (Table 2).

Table 6 lists the characteristics of the TRC strengthened slab specimens fabricated for the flexure test. As shown in Figure 7a, the region underneath the RC slab is directly strengthened by a textile grid (no lap splice) with grout; the specimen is labeled as the NA specimen. On the other hand, the LS75 specimen ($L_{LS}$ = 75 mm) and LS150 specimen ($L_{LS}$ = 150 mm) are strengthened slabs with a pair of lap-spliced textile grids. The lap-splice joint is located at the mid-span of the RC slab and the overlapped length of the grids is the same as $L_{LS}$. 

Furthermore, damaged RC slabs, i.e., failure-tested RC slab specimens in a previous study [16], were re-strengthened with the new TRC system. RC and SG-1-1 specimens in the previous study [16] were re-strengthened with the TRC system and denoted as RRC and RSG specimens, respectively. Note that the cross-sectional dimensions and material properties of the RC and SG-1-1 specimens are identical to those of the RC slab in Figure 6.

**Figure 9:**
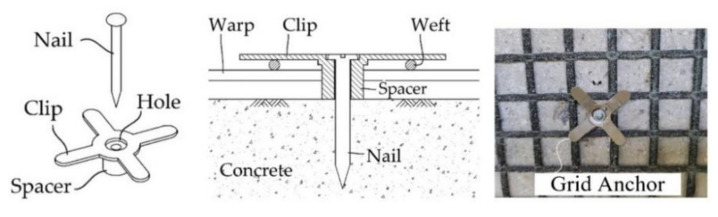
Schematics of a grid anchor system.

**Figure 10:**
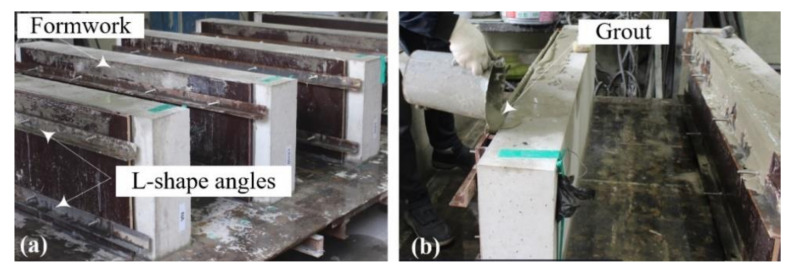
TRC-strengthened slab specimens: (**a**) assembling formwork; (**b**) grout-filling process.

Figure 8a,d show the TRC strengthening process for the RC slabs. As presented in Figure 8a, the region underneath the RC slab was ground smoothly to improve the bonding; eight 50 mm-long stainless-steel female anchors were installed in the slab. After installing the textile grid at the bottom face of the slab (Figure 8b), textile grid anchors ([24,25,26,27], Figure 9) were installed to fix the grid onto the RC slab. As shown in Figure 10a, 1800.0 mm-long and 500.0 mm-wide plywood formwork with two 20.0 mm-thick wood spacers were assembled to form a grout-filling space. Stainless steel anchor studs (diameter = 5 mm) were installed into the female-threaded anchors (Figure 7b).

In the previous study [16], the anchor studs installed to the RC slab during the strengthening work remained after removing the formwork. The failure mode of the TRC-strengthened slab specimens indicated that the anchor studs partially resist interfacial shear stress between the TRC system and the concrete substrate. 

**Figure 11:**
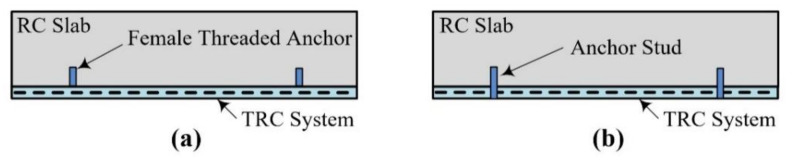
Anchor stud within TRC system: (**a**) anchors removed (NA specimen); (**b**) anchors remained (LS75, LS150, RRC, and RSG specimens).

In this study, to examine the influence of the anchor studs on the load-carrying capacity and failure mode of the TRC-strengthened slab specimens, the specimens were finished with and without the anchor studs. As illustrated in Figure 11a, the anchor studs were completely removed from the RC slab for the NA specimen. On the other hand, the anchor bolts were cut but remained after concrete curing for the LS75, LS150, RRC, and RSG specimens (Figure 11b). 

### 3.3. Test Setup

**Figure 12:**
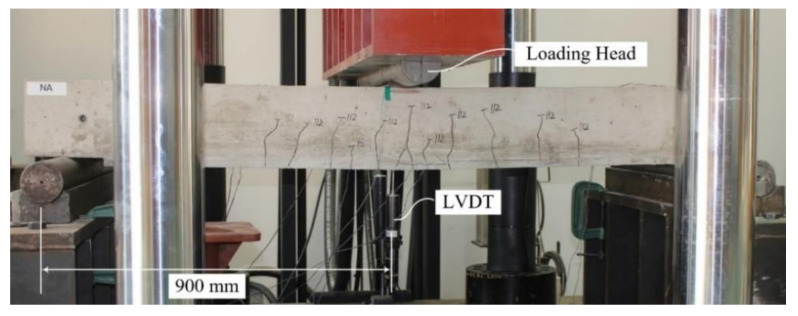
Flexural test setup and instrumentation.

Figure 12 shows a three-point bending test setup used in this study. 

## 4. Failure Test Results and Discussion

### 4.1. Load-Displacement Behavior

**Figure 13:**
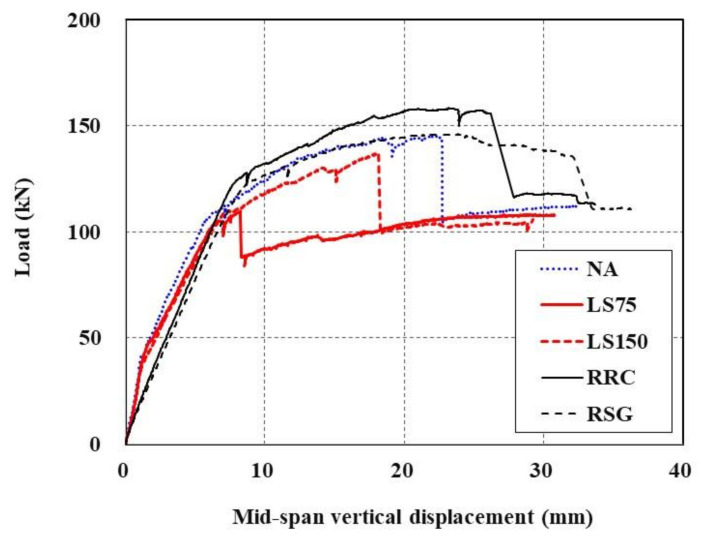
Load-displacement curves of RC slabs strengthened with TRC system.

The load-displacement curves of the slab specimens are plotted in Figure 13. As described in Section 3.2, NA, LS75, and LS150 specimens are TRC strengthened with the newly fabricated RC slabs, whereas the RRC specimen is a damaged RC slab re-strengthened with the new TRC system. Furthermore, for the RSG specimen, the damaged TRC system is completely removed from the damaged RC slab and then re-strengthened with the new TRC system.

In Figure 13, the specimens whose undamaged region underneath the RC slab is strengthened with the TRC system (NA, LS75, and LS150) exhibit load-displacement behavior in five stages. The first and second stages represent the linear behavior stage up to the initial cracking and then the load-supporting stage up to the tensile steel-reinforcement yielding, which are identical to the behaviors of the RC members. At the third stage, the specimens’ load increases to the maximum load with a certain degree of stiffness even after the yield load. The fourth stage is when the load decreases due to sudden failure following the maximum load development. Finally, the fifth stage is a section where a load similar to the yield load of the RC member is supported and only its displacement increases. In the RC members, the behavior in the fifth stage is shown after the second stage; therefore, it can be said that the third and fourth stages are characteristics that are specific to the TRC-strengthened RC members. 

On the other hand, the RRC and RSG specimens did not exhibit the concrete cracking and steel-yielding behaviors because the steel reinforcement had already yielded; therefore, the load-carrying capacity of the RRC and RSG specimens is solely due to the TRC system. 

Table 7 provides the results of a failure test for all sets of specimens. Steel yield load and ultimate load of the TRC-strengthened slab specimens with minimum textile lap-splice length (NA specimen) are increased to 127% and 134%, respectively, compared to the unstrengthened specimen (RC [16]).

In Table 8, the analytical solutions are compared with the test data (NA specimen). The analytical solutions were calculated by the procedure presented in the literature [16]. The steel yield load and ultimate load computed by the analytical procedure are 83.6% and 94.6% of the experimental results, respectively; therefore, the load-carrying capacity of RC slabs strengthened with the TRC system can be estimated by the analytical procedure for design purposes with a safety margin. 

### 4.2. Effect of Re-Strengthening with TRC System

The damaged RC slabs that experienced failure were re-strengthened with the TRC system (RRC and RSG specimens); then, a failure test was carried out on them to observe the effectiveness of the TRC strengthening for severely damaged RC slabs. Theoretically, the RRC and RSG specimens would not be able to carry any load. In the specimens that experienced failure, permanent residual displacement existed, but in the measurement process of this test, this permanent residual displacement was set as zero.

**Figure 14:**
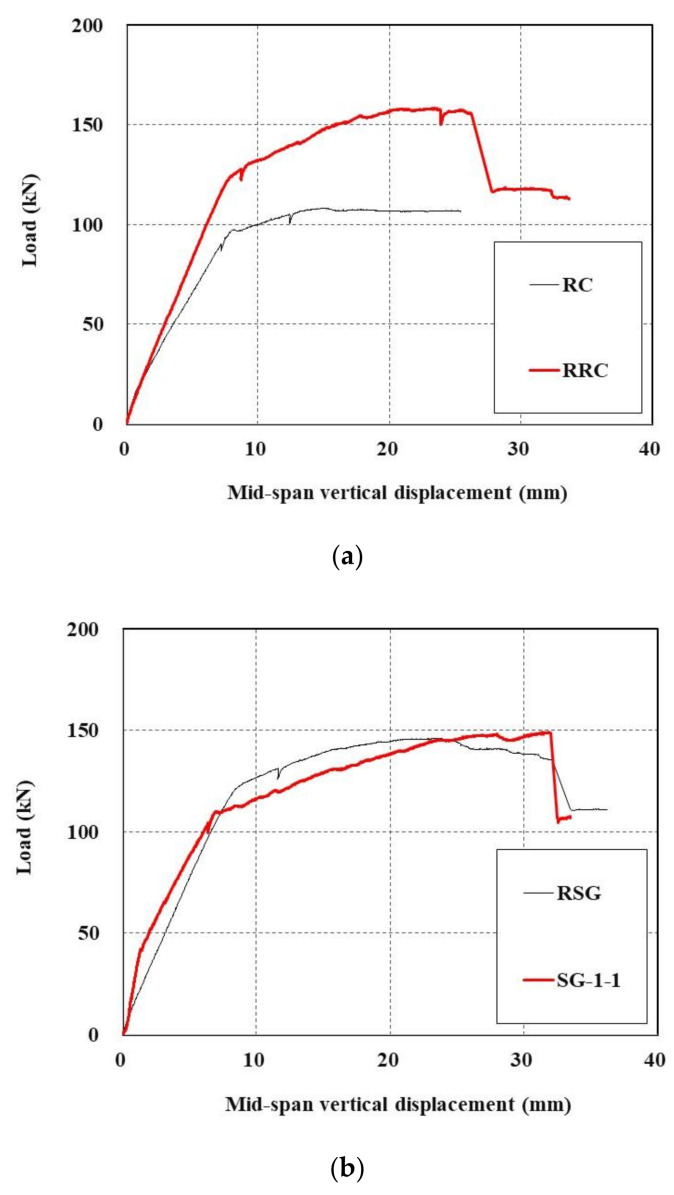
Load-displacement curves of damaged RC slabs re-strengthened with TRC system: (**a**) RC [16] and RRC; (**b**) SG-1-1 [16] and RSG.

The measured load-displacement curves are plotted in Figure 14. As shown in Figure 14a, the maximum load of the undamaged RC slab tested in [16] was 108.4 kN, and when this slab was re-strengthened (RRC), the maximum load was 158.4 kN, representing a 146.1% increase.

Moreover, in the case where the TRC-strengthened RC slab (SG-1-1 [16]) that experienced failure in Figure 14b was once again strengthened with the TRC system (RSC), displacement varied due to permanent deformation; however, considering only the maximum load, almost the same strengthening effect was found even when the specimen was restrengthened with maximum loads of 149.0 kN and 146.2 kN, respectively.

This result indicates that, with respect to the maximum load, if the tensile section of the RC slab is strengthened with the TRC system, the TRC system takes over the role of tensile steel bars as the main reinforcement; therefore, in terms of the maximum load, the desired performance will be achieved even without tensile steel reinforcements; however, because the member strengthened with the TRC system undergoes sudden failure, additional tensile steel reinforcement must be considered in order to prevent the catastrophic failure of damaged RC elements.

The maximum load of the RRC specimen is higher than that of the RSG specimen in Figure 14 because the RRC specimen has the TRC system installed on the underside surface of the slab, whereas the RRC system has its cover removed for reinforcement, resulting in different moment arm lengths.

### 4.3. Effect of Lap Splicing in TRC Strengthening

**Figure 15:**
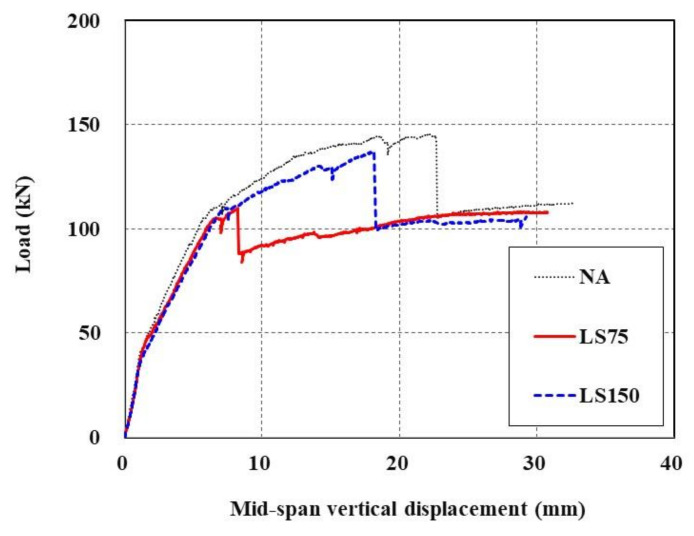
Load-displacement curves of TRC-strengthened RC slabs with (LS75 and LS150) and without lap splicing (NA).

Figure 15 shows load-displacement curves of TRC-strengthened RC slabs with and without lap splicing. As shown in Figure 15, when carbon textile grids were lap spliced at the maximum flexural moment section, they tended to exhibit the same behavior with different maximum loads in the early stage. The LS75 specimen with a relatively shorter lap-splice length failed immediately after the yield load, whereas the specimen with a relatively longer lap-splice length (LS150) displayed behavior similar to that observed when the carbon textile grid was placed with no lap splicing (NA). LS75 had the greatest maximum load, followed by LS150 and NA, and the values were 109.8 kN, 137.1 kN, and 145.4 kN, respectively. While the lap-splice length of 150 mm (LS150) represents a 100% increase from that of LS75, the maximum load of the former increased by 24.9% compared to the latter.

These results indicate that when textile grids are lap spliced, their maximum performance is reduced compared to when they are placed without lap splicing, though this may vary depending on the lap-splice length, and also that the rate of the reduction is not directly proportional to the lap-splice length; therefore, considerable attention is required when placing textile grids in the maximum flexural moment section. Although it is impossible to obtain an accurate value due to the small number of specimens, assuming that the ratio of the lap-splice length increase of 100% and the maximum load increase of 24.9% is directly proportional to the maximum load of NA (which increased by 32.4%), one can expect that maximum performance identical to that of NA without a lap splice can be achieved by increasing the lap-splice length of 75 mm by 212% to 159 mm.

### 4.4. Effect of Anchors for TRC System

**Figure 16:**
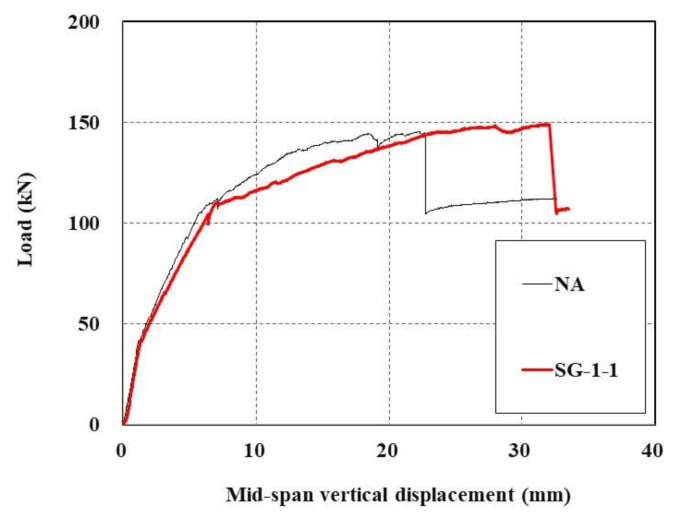
Load-displacement curves of TRC-strengthened RC slab with (SG-1-1) and without anchors (NA).

Figure 16 shows load-displacement curves representing the effect of anchoring for TRC strengthening. For the NA specimen, male anchor bolts were installed during the curing of the specimen but were completely removed after curing, and no anchors remained, while SG-1-1 [16] still had anchors remaining after curing.

The previous study [16] reported that when the RC slab was strengthened by the TRC system with anchors, the ductility was enhanced with an increase in the maximum displacement. In this test, the maximum load increased by 2.5% in specimens with anchors (SG-1-1, 149.0 kN) compared to that without anchors (NA, 145.4 kN). Further research on the effect of ductility enhancement is required as it may vary depending on the number and locations of anchors being installed.

### 4.5. Failure Pattern

**Figure 17:**
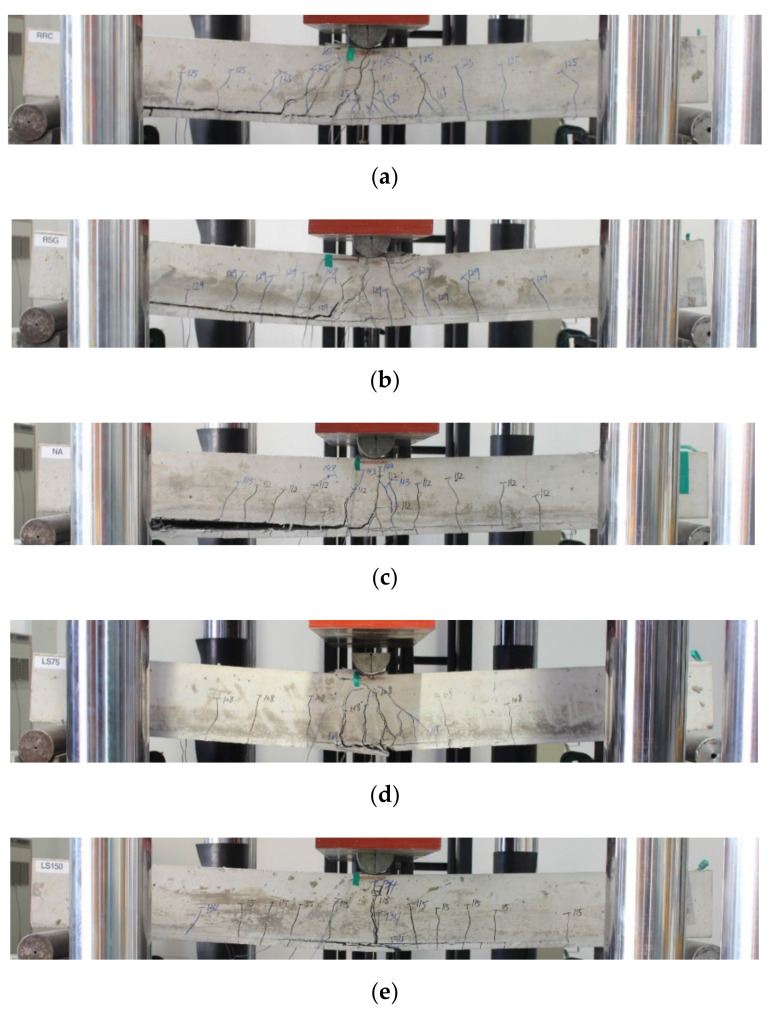
Failure patterns of specimens: (**a**) RRC, (**b**) RSG, (**c**) NA, (**d**) LS75; (**e**) LS150.

Figure 17 shows the failure patterns of the tested specimens. With the RRC and RSG specimens, only flexural cracks were observed up to about 125 kN, but these specimens finally failed due to the TRC system debonding after tensile cracks at the center had spread to the TRC-strengthened interface. As for the NA specimen, only flexural cracks were observed up to about 110 kN, but it finally failed due to the TRC system debonding after transverse cracks were observed at the TRC-strengthened interface. For the LS75 and LS150 specimens, cracks started to be observed at around 100 kN at the ends of the overlapping part of the textile grids, and then the overlapping part debonded afterwards, resulting in the specimens losing their functionality as reinforcements; therefore, the failure mode for the LS75 and LS150 specimens indicates that the location of the textile lap-splicing is a critical location for failure. 

## 5. Conclusions

This paper deals with an on-site installation method of a TRC system by grouting for strengthening of RC slabs and validation of the TRC flexural strengthening method by structural testing. In this study, undamaged and damaged RC slabs were strengthened by the TRC system. Undamaged RC slabs were strengthened with lap-spliced textile. A direct tension test was conducted to examine the tensile performance of the TRC system with lap-spliced textiles and to evaluate the minimum lap-splice length of the textile. A flexural test of full-scale RC slabs strengthened with lap-spliced textile grids was carried out to validate their structural performance.

In the direct tension test for the TRC system, sand-coated and uncoated carbon textile grids and cementitious grout were used. The results of the direct tension test confirm that the ultimate tensile strength of the TRC system was significantly affected by the textile surface treatment and the lap-splice lengths. The sand-coated textile shows better bonding performance than the uncoated textile. The minimum lap-splice length of the sand-coated textile was evaluated as 150 mm. If the lap-splice length of the textile is longer than the minimum lap-splice length of the textile, tensile stress induced in the TRC system can safely be transferred through the lap-spliced textile.

Three full-scale RC slabs were strengthened by the TRC system with and without textile lap-splicing and tested under flexure to examine the structural behavior. The load-displacement curve and load-carrying capacity of the RC slab specimens strengthened with lap-spliced textile were very similar to those without textile lap-splicing until the applied load reached the steel yield load; however, the ultimate load-carrying capacity of the RC slabs strengthened by the TRC system with textile lap-splicing decreased by at least 6% relative to that without textile lap-splicing. Furthermore, the TRC strengthened slab for which the lap-splice length of textile was 50% smaller than the minimum lap-splice length failed at the load level of steel yield. 

The failure mode of the RC slabs strengthened by the TRC system with textile lap-splicing indicates that the location of textile lap-splicing within the TRC system is a critical location for failure; therefore, considerable attention is required in the design of flexural strengthening by a TRC system with textile lap-splicing. The location of textile lap-splicing should not be at the maximum flexural moment or excessive bending curvature will occur.

In this study, damaged RC slabs where steel reinforcement completely yielded were strengthened with a TRC system. The results of a flexural test for the TRC-re-strengthened slabs indicate that the ultimate load-carrying capacity of the TRC-re-strengthened slabs is almost the same as that of an undamaged slab strengthened with the TRC system; therefore, the proposed TRC-strengthening method can effectively be used for flexural strengthening of damaged or structurally deficient RC elements. Furthermore, the total cost of the TRC strengthening for an RC slab is roughly estimated to be about 60% of that of epoxy-bonded carbon sheet strengthening [13]. Although no attempt has been made in this study, the proposed method can be applied to the shear strengthening of RC beams. For shear strengthening, a TRC jacket can be installed in the shear span of an RC beam where shear strengthening is required.

The proposed method utilizes cementitious grout as a matrix for the TRC system. Advantageous features of the proposed method of TRC installation over the conventional methods are that the proposed method can be applied to the RC structures with difficult accessibility and textile lap-splicing can be easily performed during the installation. The testing method presented in this study can be utilized to determine the minimum lap-splice length of various TRC systems in practice. 

The comparison of the test data and the analytical solutions indicate that the load-carrying capacity of RC slabs strengthened with the TRC system can be estimated by the analytical procedure for design purposes with a safety margin. Furthermore, a finite element analysis should be conducted for a more complicated RC structure strengthened with the TRC system as a future study.

A carbon grid was used as textile reinforcement in this study; however, other types of textile grids can be utilized for the proposed method of TRC installation. 

## Figures and Tables

**Table 1:** Material properties of carbon textile grids (suggested values by the manufacturers).

Textile Grid ID	Fiber (tex) ^3^	Resin	Surface Coating	Cross-Sectional Area of Textile ^4^ (mm^2^/m)	Tensile Strength (MPa)	Elastic Modulus (GPa)
Tx-1 ^1^	3200	Epoxy	Sand-coated	85	3300	220
Tx-2 ^2^	3200	Acrylate	Uncoated	85	2531	229

^1^ Q85/85-CCE-21-E4, Solidian-Kelteks, Karlovac, Croatia. ^2^ HTC 21/21-40, Hitexbau GmbH, Augsburg, Germany. ^3^ tex = Grams per kilometer of yarn. ^4^ Cross-sectional area of yarn = 1.81 mm^2^.

**Table 2:** Mixture composition of grout (Chemius Korea Ltd. Co., Seoul, Korea, unit: kg/m^3^).

Cement ^1^	Sand	Water	Silica Fume	Superplasticizer	Expansion Agent	PVA Fibers ^2^
1055.0	1130.0	142.0	42.0	8.4	99.8	0.3%

^1^ Type I Portland cement specified in ASTM C150 [21]. ^2^ Polypropylene short fibers (length = 6.0 mm).

**Table 3:** Characteristic of direct tension test specimens.

Specimen ID	Textile Grid	Textile Coating	Lap-Spliced	$L_{LS}$ (mm)	No. of Specimen
TS-0	Tx-1	Sand-coated	No	-	3
TS-75	Tx-1	Sand-coated	Yes	75	3
TS-150	Tx-1	Sand-coated	Yes	150	3
TH-0	Tx-2	Uncoated	No	-	3
TH-75	Tx-2	Uncoated	Yes	75	3
TH-150	Tx-2	Uncoated	Yes	150	3

**Table 4:** The results for direct tension tests (average values of three tests).

Specimen ID	$L_{LS}$ (mm)	$f_{cr}$ (MPa)	$f_{fu}$ (MPa)	$f_{fu}$ Gain (%)	$\varepsilon_{fu}$
TS-0	-	2.8	2112.5(93.1)	100	1.912(0.182)
TS-75	75	2.4	1689.3(109.1)	80.0	1.447(0.114)
TS-150	150	3.2	2169.2(90.6)	102.7	1.716(0.230)
TH-0	-	3.7	923.5(250.6)	100	0.019(0.006)
TH-75	75	4.1	961.3(35.6)	104.1	0.028(0.006)
TH-150	150	2.8	912.9(118.7)	98.9	0.029(0.004)

Note: value in ( ) is a standard deviation of test data.

**Table 5:** Mix composition and design strength of ready-mixed concrete (unit: kg/m^3^).

Cement	Water	Fly Ash	GGBS	Sand	Coarse Aggregate ^1^	Superplasticizer	Design Strength
263	167	56	56	828	934	2.63	27 MPa

^1^ Maximum grain size = 25 mm.

**Table 6:** Characteristic of TRC-strengthened slab specimens for flexural test.

Specimen ID	Slab Condition	Textile Lap-Spliced	Anchor Studs	Re-Strengthened	No. of Specimens
NA	New	-	Removed	-	1
LS75	New	Yes	Remained	-	1
LS150	New	Yes	Remained	-	1
RRC	Damaged	-	Remained	Yes	1
RSG	Damaged	-	Remained	Yes	1

**Table 7:** Results of failure test for RC slab specimens.

Specimen ID	Steel Yield		Ultimate Stage		Load Gain (%)	
	Load (kN)	Disp. (mm)	Load (kN)	Disp. (mm)	At Steel Yield	At Ultimate Stage
RC [16]	82.5	6.5	108.4	15.0	100.0	100.0
NA	105.4	6.0	145.4	22.4	127.7	134.1
LS75	103.2	6.5	109.8	8.5	125.1	101.3
LS150	96.5	6.1	137.1	18.2	117.0	126.5
RRC	-	-	158.4	22.8	-	146.1
RSG	-	-	146.2	23.2	-	134.9

Note: Disp. = mid span vertical displacement.

**Table 8:** Comparison of test data and analytical solutions.

Analytical Solutions				Analytical Solutions/Test Data (%)			
Steel Yield		Ultimate Stage		Steel Yield		Ultimate Stage	
Load (kN)	Disp. (mm)	Load (kN)	Disp. (mm)	Load (kN)	Disp. (mm)	Load (kN)	Disp. (mm)
88.1	4.8	137.6	18.3	83.6	80.0	94.6	81.7

## Data Availability

The data presented in this study are available on request from the corresponding author.
